# METscout: a pathfinder exploring the landscape of metabolites, enzymes and transporters

**DOI:** 10.1093/nar/gks886

**Published:** 2012-09-27

**Authors:** Lars Geffers, Benjamin Tetzlaff, Xiao Cui, Jun Yan, Gregor Eichele

**Affiliations:** ^1^Department of Genes and Behavior, Max Planck Institute for Biophysical Chemistry, ^2^Anwendungs- und Informationssysteme, Gesellschaft für wissenschaftliche Datenverarbeitung mbH Göttingen, Am Faßberg 11, 37077 Göttingen, Germany and ^3^Functional Genomics Group, CAS-MPG Partner Institute for Computational Biology, 320 Yue Yang Road, 200031 Shanghai, China

## Abstract

METscout (http://metscout.mpg.de) brings together metabolism and gene expression landscapes. It is a MySQL relational database linking biochemical pathway information with 3D patterns of gene expression determined by robotic *in situ* hybridization in the E14.5 mouse embryo. The sites of expression of ∼1500 metabolic enzymes and of ∼350 solute carriers (SLCs) were included and are accessible as single cell resolution images and in the form of semi-quantitative image abstractions. METscout provides several graphical web-interfaces allowing navigation through complex anatomical and metabolic information. Specifically, the database shows where in the organism each of the many metabolic reactions take place and where SLCs transport metabolites. To link enzymatic reactions and transport, the KEGG metabolic reaction network was extended to include metabolite transport. This network in conjunction with spatial expression pattern of the network genes allows for a tracing of metabolic reactions and transport processes across the entire body of the embryo.

## INTRODUCTION

Digital atlases of spatiotemporal gene expression patterns have been generated for vertebrates (1) and invertebrates (2). The transcriptome atlases for the mouse brain (3) and the E14.5 mouse embryo (4,5) contain expression patterns for nearly 20 000 genes created by subjecting serial sections of the brain or embryo to robotic *in situ* hybridization (ISH). Although expression patterns are determined section-by-section, the number of sections used is sufficient to create for each gene a spatial expression volume (3–5). This completeness in spatial coverage in combination with the standardized methods used to create and annotate the data, the genomic scale and the web accessibility, make brain and embryo atlases widely used resources. Although efforts have been undertaken to exploit such atlases for addressing biological questions on an -omic scale (6–8), these resources are thought to be underutilized (9) and have much additional potential that is barely used. In part, this is because an expression atlas *per se* contains only one type of data and it would be useful to generate databases in which data of different modalities can be used in combinatorial queries. Toward this end, we have developed the ‘METscout’ (Metabolites Enzymes and Transporters) database in which we bring together metabolic pathway maps with the expression patterns of ∼1500 metabolic enzyme genes and ∼350 metabolite transporter genes. The expression pattern of >300 of these genes was determined *de novo* for METscout whereas the rest was from Genepaint.org and Eurexpress.org. METscout is currently based on expression data for the E14.5 mouse embryo but tissue anatomy and histology and metabolic needs at E14.5 resemble those of postnatal stages.

Metabolic pathways used in METscout are taken from the Kyoto Encyclopaedia of Genes and Genomes (KEGG) (10), a comprehensive repository of well-curated metabolic pathway maps. Metabolic pathways and networks represent metabolites as nodes that are connected by edges symbolizing chemical reactions. Classical biochemistry and comparative genome annotation have led to assignment of specific enzymes to many of the edges. METscout integrates metabolic transporters into the KEGG network. This incorporation of transporters is an important addition because it opens the possibility for understanding the exchange of metabolites within and between tissues and organs.

METscout rapidly retrieves collections of genes that are associated with a particular metabolic pathway, exhibit the same enzymatic activity, are expressed in the same organ or interact with the same metabolites. For each collection, a sortable matrix of images (or image abstraction icons) is generated which can then be visually inspected using a number of user-friendly representations that facilitate comparison of genes within a collection. Because all expression sites of metabolic enzymes and transporters are represented in METscout, the user can undertake a pathway walk, starting from e.g. a metabolite and then walk along the cognate metabolic pathways from one reaction to the next and potentially even exit the expression site passing through gates provided by transporters.

## DATABASE CONTENT

METscout is a gene-centric database with an almost full coverage of metabolic network components of mouse. Relevant genes were selected on the basis that they are either present in one of the mouse-specific metabolic pathways in KEGG (11), are orthologous to genes in the *Homo sapiens* metabolic reconstruction of BiGG (12), are members of the ABC-transporter family or of the solute carrier (SLC) superfamily. Genes were grouped in three categories: (i) enzyme encoding genes with EC-numbers and/or metabolic reactions in KEGG; (ii) transporter encoding genes of the SLC superfamily; and (iii) other genes that are not yet formally linked to the metabolic network in METscout such as the ABC-transporter family. Each gene in METscout is represented by a single dataset that is uniquely identified by its NCBI Gene ID. This Gene ID is the primary key for all other gene-related information retrieved from external resources such as NCBI, GenePaint or KEGG. A dataset consists of a gene information summary, experimental ISH data and the textual annotation of gene expression. In addition, each dataset was matched with microarray expression data from whole E14.5 embryos (NCBI GEO; GSM140700, GSM140701). For datasets of enzymes the metabolic relationship to pathways, reactions and compounds was deduced from the formal description of KEGG pathway maps specific for mouse (publicly available files in KGML format). Datasets of transporters were related to pathways and reactions based on compounds. Solutes transported by SLCs were obtained from the literature (13,14).

## DATABASE FUNCTIONALITY

### Queries

Queries can be entered in the topic and query fields. First the desired topic is selected from the drop-down menu (Gene, EC-Number, Reaction, Compound, Pathway or Organ). The ‘All’ button opens an inventory of all items available for the selected topic such as gene symbols, EC-numbers, etc. Alternatively, a search term can directly be entered into the query field. This is aided by an auto-complete function suggesting eligible items that contain the entered string at any position. The ‘Go’ button then launches the query and will bring up the Result Page.

### Quick Start

**Figure 1.**
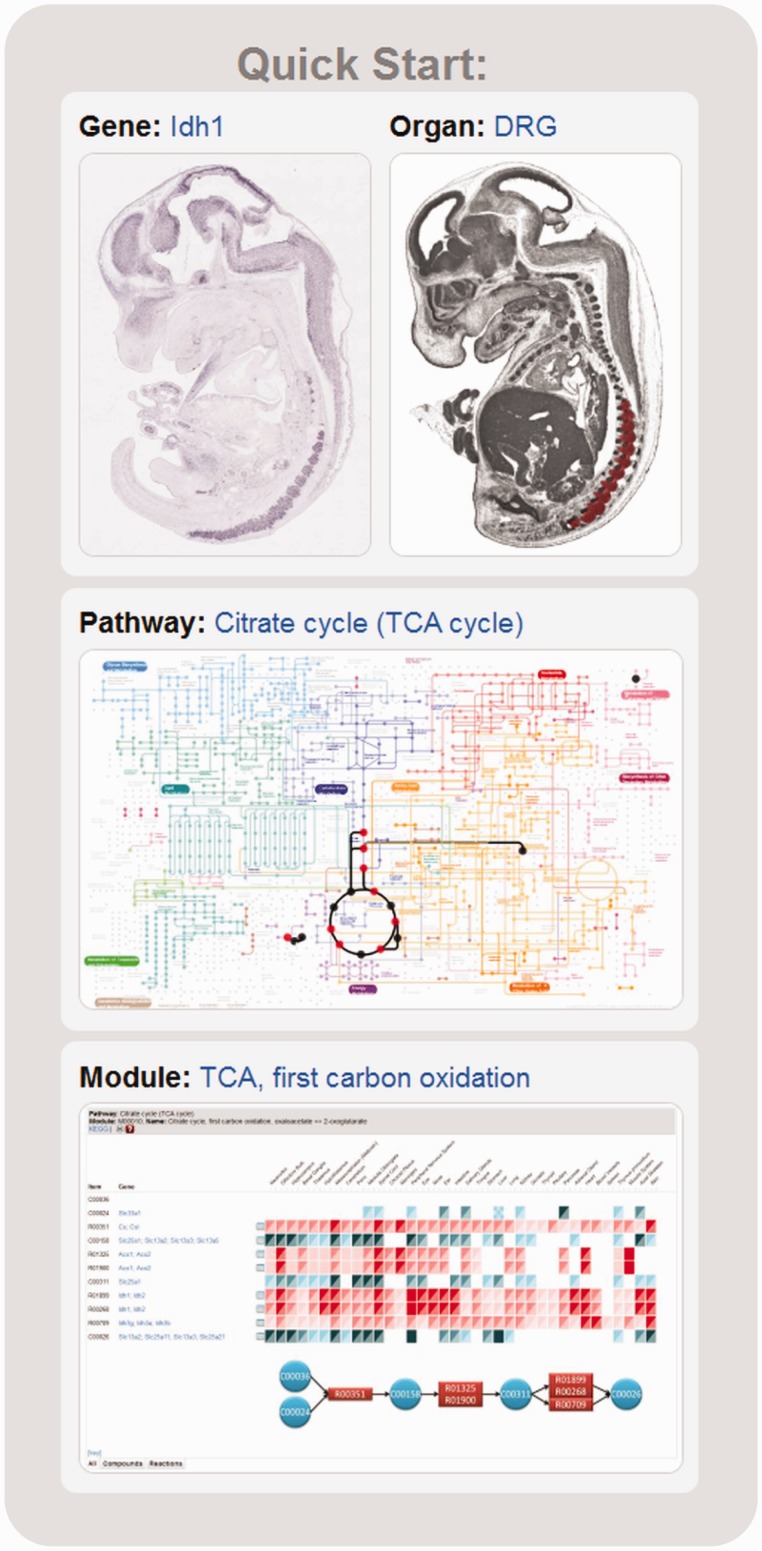
Quick Start. METscout offers a panel with randomly changing content each time the home page reloads. The user can explore four different aspects of METscout functionality starting with a gene (*Idh1*, top left) contained in a particular pathway (TCA cycle, center) and module (TCA first carbon oxidation, bottom). Another entry point is an organ where the selected *Idh1* gene is expressed (dorsal root ganglia, DRG, top right).

As an alternative entry point the home page of the METscout database offers a Quick Start panel with randomly changing content. This is meant to allow the user to instantly explore different aspects of database functionality within the context of a particular metabolic pathway such as the TCA cycle (Figure 1). The user can choose from four different topics selecting a gene from the pathway, an organ where this gene is actually expressed, the pathway itself or a module which is defined as a subset of reactions within the pathway. Each query will bring up the Result Page though choosing a module will additionally open the ModuleView window.

### Result page

**Figure 2.**
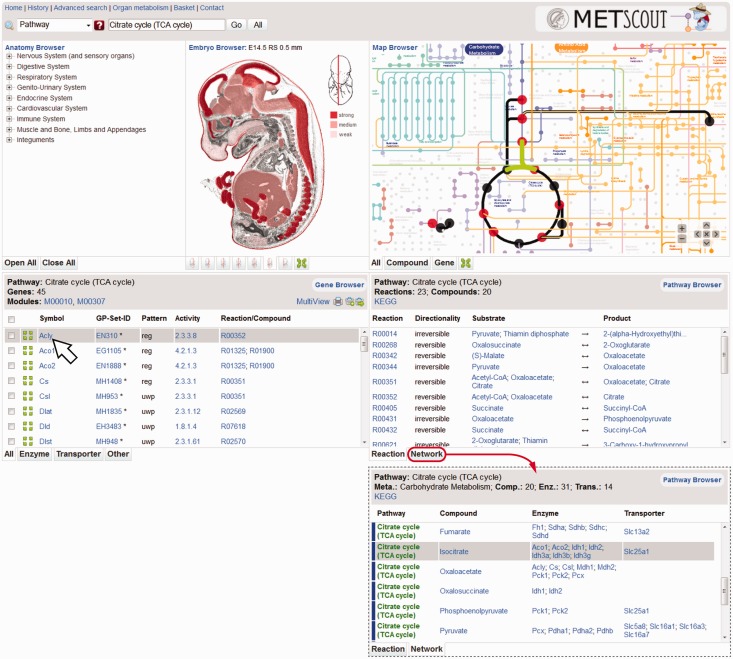
Result Page showing the result of a pathway search for ‘Citrate Cycle (TCA cycle)’. Results of the query are presented in five distinct panels located below the query field: Anatomy Browser (top left), Embryo Browser (top center), Map Browser (top right), Gene Browser (bottom left) and Pathway Browser (bottom right). Compounds (nodes) and reactions (edges) of this pathway are highlighted in the Map Browser. Red nodes represent compounds for which a specific SLC exists. The Gene Browser provides a list of all genes functionally associated with the TCA cycle whereas the Pathway Browser provides a list of pathway-specific reactions (‘Reaction’ tab) and compounds (‘Network’ tab). Pointing at any gene symbol in any of the lists simultaneously highlights its expression pattern in the Embryo Browser (colored areas) and its metabolic activity in the Map Browser (green).

The result page is composed of five browser panels: Anatomy Browser, Embryo Browser, Map Browser, Gene Browser and Pathway Browser (Figure 2). The term ‘browser’ signifies that in each of the panels new queries can be initiated by following text links (blue) or graphical links that are indicated by a tooltip or highlighted in green upon mouse-over. Whereas the upper browser panels illustrate the query result, the lower panels list the corresponding datasets (Gene Browser) and summarize related reactions and compounds (Pathway Browser). The lower panels are headed by gray info boxes that restate the query argument and provide additional information, such as statistics, as well as links and tools.

The *Gene Browser* (Figure 2, bottom left) provides a list of all genes that are associated with the selected pathway either by encoding an enzymatic activity or a transporter for any of the pathway’s compounds. For each gene the list provides several types of information: (i) Symbol, official gene symbol linking to the corresponding METscout dataset; (ii) GP Set ID, link to the original ISH dataset in GenePaint; (iii) Pattern, indicates with respect to the whole embryo whether gene expression is regional (reg), ubiquitous with pattern (uwp), ubiquitous (ubi) or not detected (nd); (iv) Activity, links to corresponding EC-numbers in case of enzymes and states ‘transport’ in case of SLCs; and (v) Reaction/Compound, lists and links the actual reactions/compounds catalysed/transported by the gene product in the context of the original query. Check boxes are used to select all or a subset of listed genes in order to view their expression patterns side by side (see Section ‘MultiView’) or for collection and storage in a basket (see Section ‘Basket’). The gene list can be filtered (Enzyme, Transporter, Other) by selection of the appropriate tabs found at the bottom. The layout of the Gene Browser is the same for all query types except when the result is a single gene. In this case, thumbnails of the corresponding ISH dataset are directly shown instead of a table and an info button links to a new window providing detailed gene information including microarray data of whole E14.5 mouse embryo.

The *Pathway Browser* (Figure 2, bottom right) also has tabs at the bottom. The ‘Reaction’ tab shows all reactions associated with the query (TCA cycle). The direction of a reaction is indicated and the main substrate and product names are given. Reactions and compounds can be selected to initiate new queries. Choosing the ‘Network’ tab displays a table with four interdependent columns (Pathway, Compound, Enzyme and Transporter) with each row representing a pathway-compound combination matched against the query argument. In the example provided (Figure 2) the pathway is the ‘TCA cycle’ and all compounds of this pathway are listed. However, if a single gene is the subject of a query it may bring up several pathways-compound combinations because a particular gene product may interact with several compounds that belong to different pathways. Genes listed under ‘Enzyme’ and ‘Transporter’ encode for gene products that can potentially interact with the compound listed in the same row. Since all items in this table are links usable to start off a new query, the ‘Network’ listing allows one to follow a metabolic process along several pathways as will be described in the Section ‘Pathway Walking’ below. The layout of the Pathway Browser is the same for all query types with exception of the organ query where a list appears that uses gene expression to compute the number of reactions of each pathway that may take place in the selected organ.

The *Map Browser* (Figure 2, top right) is directly adapted from the KEGG Atlas (11) and implemented as an interactive Shockwave Flash Movie (swf). Compounds (nodes) and reactions (edges) are color-coded delineating different metabolisms within the network. Cartouches of the same color name the metabolisms (e.g. amino acid metabolism) whereas colored text represents the different subtypes of metabolisms (e.g. tryptophan metabolism). All nodes and edges associated with the query argument are marked in black or, alternatively, nodes are in red in case they represent a Slc-transportable compound. If the user points to a gene symbol, a reaction or a compound in the Gene Browser or Pathway Browser, the Map Browser reacts by highlighting relevant network components in green. The KEGG-derived Map Browser is a comprehensive but for reason of clarity an incomplete representation of the metabolic network (11). Therefore, some of the reactions or compounds found in the Gene or Pathway Browsers may not be represented in the Map Browser. In the tool tip this is indicated by ‘NOT IN MAP’. Nonetheless, the map allows a rapid assessment of the metabolic context in which a gene or compound is engaged. Using the tabs at the bottom of the panel one can filter the map to display either only labeled nodes (Compound) or edges (Gene). The map can be toggled between page filling size and standard size using the maximize tab. It can be zoomed in and panned using the pop-up menu inside the Flash application or using the keyboard (plus, minus, arrow keys).

The *Embryo Browser* (Figure 2, top center) provides schemes of the embryo sections and expression sites (red color hues). It consists of seven representative sagittal histological sections of an E14.5 mouse embryo which collectively represent all 96 anatomical structures (‘organs’) for which gene expression had been annotated in Genepaint. Individual planes of sectioning can be selected from the icons tabs at the bottom in which a red vertical line indicates the section position. Maximizing, zooming and panning are as described for the Map Browser. Different shades of red seen in any of the 96 organs reflect the relative signal intensity of a particular gene expression pattern. Strong expression is represented by dark red (see legend right of the section). Regions that are not highlighted by a red hue are devoid of detectable expression. Whenever a gene symbol is selected or pointed at (e.g. arrow in Figure 2), its stylized expression pattern appears in the Embryo Browser. This capability of METscout is extremely useful to obtain a rapid review of the expression patterns of multiple genes. Moreover, if an organ is queried, the Embryo Browser will highlight in red the selected organ in the most representative section.

The *Anatomy Browser* (Figure 2, top left) is a hierarchical tree with a total of 126 structures and 96 leaf structures termed ‘organs’. Using the tabs at the bottom of the panel the tree can be expanded or collapsed. Organ queries can be initiated by picking a term in the tree. In contrast to the Embryo Browser, the Anatomy Browser includes also upper tier structures such as the ‘Endocrine System’ that consists of multiple organs (thyroid, pituitary, pancreas and adrenal gland). This is useful for those primarily interested in the expression profiles of whole organ systems rather than in individual organs. The Anatomy Browser documents expression of a particular gene selected with regard to strength and pattern. Expression is represented by numbers ranging from ‘NONE’ (not detected) to ‘3’ (strong) and letters (u: ubiquitous; r: regional; s: scattered). To allow for a direct comparison between annotation and the original ISH data, the associated icons symbolizing expression directly link to a representative ISH image.

## TOOLS

To generate an integrating competence in METscout several tools were programmed which are (i) the MultiView comparing the expression patterns of gene lists such as all genes in a pathway; (ii) the ModuleView for showing the expression patterns of the KEGG-defined pathway modules; and (iii) the Basket in which user-selected search results can be temporarily stored.

### MultiView

The MultiView function is activated from a link in the gray info box of the Gene Browser. To view expression data for multiple genes, all appropriate boxes are checked and the query is submitted using the MultiView link. This opens a separate window with three tabs to study the selected genes in the AnnotationView (default), the ImageView or the MapView.

**Figure 3.**
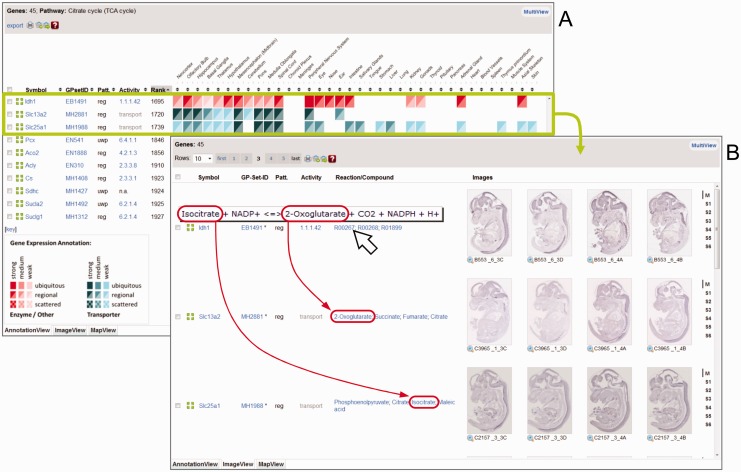
MultiView of genes from the TCA cycle. TCA cycle genes were submitted to MultiView that graphically represents them in three different modes (selectable from tabs at the bottom of the MultiView panel). AnnotationView (**A**, default) consists of a sortable table of gene-associated information and an iconic representation of gene expression strength and pattern (key provided below table). The ImageView (**B**) shows representative ISH data for each of the genes of the AnnotationView. The MapView (not shown) highlights gene product activities in the metabolic map. Genes in AnnotationView can be ranked according to expression pattern similarity using the arrowheads of the ‘Rank’ column. In case of the TCA cycle *Idh1* (an enzyme converting isocitrate into 2-oxogluterate) is ranked next to *Slc13a2* and *Slc25a1* (boxed in green), the transporters translocating its substrate and product. The ImageView shows that the expression of *Idh1*, *Slc13a2* and *Slc25a1* overlaps prominently in the nervous system.

The *AnnotationView* (Figure 3A) provides a comprehensive way to compare gene expression patterns of many genes such as all genes of the TCA cycle. The data are organized into a table where rows represent genes and columns gene attributes, such as the gene expression scores for the individual anatomical sites. For sake of clarity, the ∼100 anatomical terms of the original anatomical tree were reduced to 36 selected terms still representing all aspects of anatomy (e.g. the terms ‘lens’, ‘retina’, ‘cornea’, ‘optical nerve’ are represented by their parent structure ‘eye’). Gene expression is graphically represented with symbols similar to those used in the Anatomy Browser (see Figure 3 for key, blank fields mean that no expression was detected). Clicking any of the symbols in the table will bring up a new window with a representative ISH image of the corresponding gene showing expression in the selected structure. A powerful functionality of the AnnotationView table is that each column can be sorted in any direction by repeatedly pressing the arrows in the table head. All genes in the database have been ranked in a pre-computed hierarchical clustering analysis of their gene expression patterns. Sorting according to the ‘Rank’ column brings similar patterns next to each other (Figure 3, green box) that can then be inspected with ImageView.

The *ImageView* (Figure 3B), activated by the appropriate Tab, displays genes submitted to MultiView in the same order as in AnnotationView. Resorting the table in AnnotationView will automatically change the order of genes in ImageView accordingly. For each gene, representative ISH images are shown as thumbnails. As a default, medial sections (M) of the embryo are displayed, but other sectioning planes (S1, S2, S3, S4, S5 or S6) can be retrieved from a menu at the right side of each thumbnail strip. Thumbnails can be enlarged by clicking the images or even be inspected at full resolution using the magnifying glass below the thumbnail. In order to accelerate page loading only three rows of genes are initially shown. However, the drop-down menu at the top of the ImageView page allows for the simultaneous display of up to 100 rows on a single page. Instead of having one large page, the menu also affords a multipage representation. To relate genes back to metabolism, the MapView tab is used.

In *MapView* all genes submitted to MultiView are projected onto the metabolic map. In contrast to the Map Browser all reactions and compounds associated with the submitted genes are highlighted in the map regardless of the initial query context. MapView can be efficiently used to check whether a selection of genes could be part of a common metabolic network.

### ModuleView

**Figure 4.**
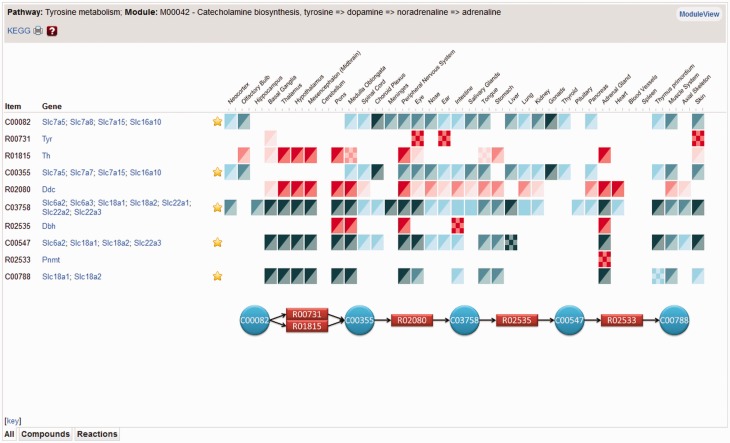
ModuleView. The module ‘catecholamine biosynthesis’ schematically shown at the bottom is modified from KEGG (11) and consists of several reactions interconverting tyrosine to adrenaline. Expression information for module enzymes (red icons) and putative transporters (blue icons) is shown for each of 36 organs thereby providing a synopsis to assess in which organ the ‘catecholamine biosynthesis’ module is likely to be realized. In the case a reaction or transport is mediated by multiple gene products, ModuleView pools all expression patterns into a single profile that can, however, be expanded using the star icon left to the expression profile.

A variant of the MultiView function is used for displaying metabolic pathway modules which are defined by KEGG as ‘tighter functional units’. If a chosen pathway has any number of these modules, METscout will display corresponding links in the Gene Browser. Following such a link will lead to a new window (ModuleView, Figure 4) that lists in the ‘Item’ column all compounds and reactions of the module (chemical names and reaction equations are obtained upon mouse-over or by the ‘key’ link). Compounds are related to genes that encode the cognate transporters, and reactions are related to genes that encode corresponding enzymatic activities.

Figure 4 shows the KEGG ‘catecholamine biosynthesis’ module within the tyrosine pathway (tyrosine ⇒ dopamine ⇒ noradrenaline ⇒ adrenaline). Expression strength and pattern is represented by icons (Figure 3 for definition of icons). Graded expression strength of enzymes (red to pink) and transporters (dark blue to turquois) allow one to easily identify organs in which this module is present, absent or partially realized. Enzymatic activities R01815 (tyrosine hydroxylase, Th), R02080 (dopamine decarboxylase, Ddc), R02535 (dopamine beta hydroxylase, Dbh) are prominently expressed in the nervous system and in the adrenal gland. In thalamus, hypothalamus and mesencephalon enzymes catalysing R01815, R02080 are expressed leading to the production of C03758 (dopamine). The next catalytic step (R02535) leading to C00547 (noradrenaline) takes place in pons and medulla, the site of noradrenergic cell groups including the *locus coeruleus*. To its fullest extent, the module is realized in the adrenal gland which also carries out R02533 (phenylethanolamine-N-methyltransferase, Pnmt) leading to adrenaline (C00788). Note that transporters required for the transmembrane translocation of the module compounds are present in those organs that strongly express the module enzymes. It should be emphasized that the ‘Gene’ column pools all enzymes and transporters that can potentially mediate catalysis or movement. The star icon is a link to a MultiView window in which expression pattern in the pool can be viewed individually, either as icons or as ISH images. The latter are required to assess which of transporters co-localize with module enzymes.

### Basket

In order to compare datasets resulting from independent queries METscout offers a basket system in Gene Browser, AnnotationView and ImageView. A shopping basket icon with a plus sign is for adding items and a basket with an arrow allows for reviewing the basket’s content. Datasets in the basket can be entirely or individually checked. Options in the basket are applying the MultiView function (MultiView), passing the selected content as a new query to METscout (Browser), adding datasets by entering gene symbols manually (Custom), exporting the gene list as a CSV file (Export), or selective deletion of the content from the basket (basket icon with minus). After 3 h being inactive or when the session is terminated, the basket automatically clears.

## PATHWAY WALKING

A hallmark of METscout is navigation within the combined anatomical and biochemical spaces. The ‘Network’ tab in the Pathway Browser serves as a starting point for systematically walking through these spaces as we illustrate with catecholamine biosynthesis that starts from phenylalanine, an essential amino acid that arises from outside of mouse metabolism. Phenylalanine (Phe) is entered in the query field and leads to a Result Page in which Phe appears as red dot in the Map Browser indicating that Phe is a solute for a transporter. The Phe node lies within the ‘Amino Acid Metabolism’ domain (yellow). Two black edges initiate from Phe leading to tyrosine and phenylpyruvate nodes. The Pathway Browser (Network tab) shows that Phe plays a role in three pathways whose constituent enzymes that interact with Phe are listed. SLCs that can transport Phe are also listed. Upon mouse-over of enzymes and transporter gene symbols, their schematic expression patterns appear in the Embryo Browser as marked color fields and in the Map Browser as highlighted edges (enzymes) or nodes (transporters). As becomes apparent in the Map Browser, tyrosine (Tyr) is the only compound that links Phe metabolism to catecholamine biosynthesis and that this link is provided by phenylalanine hydroxylase, a liver enzyme as revealed by the Embryo Browser. Other enzymes in Phe containing pathways (e.g. Got1, Got2, etc.) do not catalyse reactions producing a catecholamine precursor and are thus disregarded in this pathway walking example. Following the Pah link brings up the Result Page in which the Gene Browser shows ISH data illustrating the highly liver-specific expression of the *Pah* gene.

The pathway walk is continued in the Pathway Browser by searching for Pah (highlighted in green) products. In the case of Pah the only product is Tyr and upon following its link five pathways appear that all use Tyr. The Map Browser shows that among all enzymes listed in the Pathway Browser only tyrosine hydroxylase (Th) feeds into catecholamine biosynthesis. The Embryo Browser reveals that Th is expressed the nervous system and adrenal gland but not in liver, as was the case for Pah. This suggests that Tyr produced in liver and consumed in the nervous system (or adrenal) needs to undergo transport. Tyr transport is mediated by Slc7a5; Slc7a8; Slc7a15; Slc16a10 (see column ‘Transporter’ in Pathway Browser). Slc16a10 known to transport Tyr, Phe, L-DOPA and tryptophan is expressed in liver and in the central nervous system (see also Gene Browser for detailed expression of *Slc16a10*) and therefore may provide a route for moving Tyr out of the liver into the brain. Pathway walking can now be continued leading from Tyr to L-DOPA etc. This example illustrates how METscout provides intuitive tools that seamlessly link enzymatic reactions and transport processes to the sites of expression of the underlying genes.

## CONCLUSION AND FUTURE DIRECTIONS

METscout, for the first time, allows one to explore the spatial organization of metabolic pathways and metabolite transport in the whole organism in an interactive fashion on the internet. This interactivity is made possible because the database contains the expression patterns of nearly all metabolic enzymes and SLCs that connect with each other through active links. A second asset of METscout is the incorporation of a combined metabolism-transport network. In the future pathway walking as described above for a specific example should be automated. This would be a prerequisite to comprehensively examine inter-organ metabolite transport, to uncover how metabolic processes are spatially segregated, and to search for missing components of metabolic networks. At a later stage METscout will have to incorporate metabolite concentrations and rates of enzymes and transport processes. This information would endow the database with a dynamic representation of spatiotemporal metabolism but it will be a challenge to determine enzyme- and transport kinetics *in vivo*.

METscout also provides a basic framework to investigate whether and how pathways may change under pathological conditions. For example, mutations in metabolic enzymes or transporters are long known to impair progression of enzymatic reactions and divert metabolites into unwanted pathways. How this perturbation propagates throughout the network could be explored with METscout. Because the database also contains expression patterns of orphan SLCs it is conceivable that their expression patterns can be utilized to infer their cognate solute and their position within the metabolic network by comparing orphan expression patterns with that of all known components of METscout. Last but not least, METscout provides a novel educational tool to navigate through metabolic charts and does this with the added knowledge of where enzymes and transporters are actually expressed.

## TECHNICAL BACKGROUND

The METscout web-interface runs on a Linux platform with mySQL 5.1 as the database engine. The web-interface was developed in HTML, PHP, JavaScript, Ajax and ActionScript 3 (Flash). All images are stored as JPGs in two different resolutions (thumbnail and medium-sized preview) in the file system. In addition, full resolution images (Kodak FPX) can be accessed via a Java Applet from a dedicated Zoom-Server. The web server and all parts of the database including the Zoom-Server are hosted at the Gesellschaft für wissenschaftliche Datenverarbeitung mbH Göttingen, Germany.

## DATABASE AVAILABILITY

The METscout web-interface is available at http://metscout.mpg.de (10 September 2012, date last accessed). Using the website and database is free and does not require registration. The web-interface is optimized for the cross-platform Mozilla Firefox Browser (>14.0). For optimal user experience swf and Java Plug-In should be installed and JavaScript needs to be enabled. This article should be cited when using data retrieved from METscout (e.g. gene expression annotation) for downstream computational applications or image publication.

## FUNDING

Research was conducted at the Max Planck Institute for Biophysical Chemistry, supported by the Max Planck Society (to G.E., L.G. and B.T.), and at the CAS-MPG Partner Institute for Computational Biology, supported by the Max Planck Society and Chinese Academy of Sciences (to X.C. and J.Y.). Funding for open access charge: Max Planck Society.

*Conflict of interest statement*. None declared.
